# All-Dielectric Metasurface-Based Beam Splitter with Arbitrary Splitting Ratio

**DOI:** 10.3390/nano11051137

**Published:** 2021-04-28

**Authors:** Xueyu Chen, Haijian Zou, Mingyang Su, Linwei Tang, Chaofeng Wang, Shuqing Chen, Chenliang Su, Ying Li

**Affiliations:** International Collaborative Laboratory of 2D Materials for Optoelectronics Science & Technology of Ministry of Education, Engineering Technology Research Center for 2D Material Information Function Devices and Systems of Guangdong Province, Institute of Microscale Optoelectronics, Shenzhen University, Shenzhen 518060, China; chenxueyu2020@email.szu.edu.cn (X.C.); 1800282020@email.szu.edu.cn (H.Z.); sumingyang@email.szu.edu.cn (M.S.); 2016170127@email.szu.edu.cn (L.T.); 2172281579@email.szu.edu.cn (C.W.); shuqingchen@szu.edu.cn (S.C.); chmsuc@szu.edu.cn (C.S.)

**Keywords:** beam splitter, metasurface, nano-ring, integrated optics

## Abstract

The development of optical systems is heading to multi-branch circuit design and miniaturization. A beam splitter is a common device for dividing an incident beam into two separate beams. Conventional beam splitters are constructed using coated prisms or glass plate. Their bulky size, right-angled output direction, and fixed splitting ratio greatly limit the design of optical arrangement and also hinder the system integration. Here, an all-dielectric metasurface composed of symmetric nano-rings as a beam splitter are designed by Finite-Difference Time-Domain method. By changing the inner and outer radiuses of the nano-rings, the wavefront phase of the emergence beam can be adjusted to form a phase gradient, and the incident beam of arbitrary polarization is divided into two beams according to the designed transmittance and angle. The initial phase of the emergence beam can be changed by adjusting the refractive index of the substrate or adding the silicon film to the substrate, and the splitting ratio can be adjusted from 0.5:1 to 1:1. The simulation demonstrates that the metasurface-based beam splitter is independent of polarization and the power efficiency is over 92% with a compact area of 33.6 μm × 33.6 μm. This compact metasurface-based beam splitter has promising potential for enabling new types of compact optical systems and advancing metasurface-based functional integrated photonic applications.

## 1. Introduction

The pursuit of photonic integration systems drives the present study of integrated photonics. With the growing maturity of nanomachining technology, it makes possible to build increasingly sophisticated and complex optical systems in a small size and requires their components to be miniaturized and multi-functionalized. A beam splitter (BS) is a critical component for distributing and combining beam paths in many optical systems. In regular optical systems, the most common cube BS are constructed using two typically right angle prisms to split incident beam into two equal beams vertically [1]. It can also be used in reverse to combine two different beams into a single one. Furthermore, silicon beam splitters are used for MZM, splitting, and combining in waveguide systems [2,3,4]. However, the splitting effect relies on the birefringence of heavy and solid glass construction. It makes normal BS hard to integrate into compact optical systems. Some flat-type BSs like plate BS [5], grating BS [6], and dichroic BS [7] have been previously demonstrated. Although their sizes are significantly smaller than cube BS, these BSs are still not suitable for integration owing to their fixed splitter ratio, different lengths of beam paths, and expensive materials.

Metasurface, composed of a periodic arrangement of artificial microstructure that is smaller than the wavelength of light, can manipulate electromagnetic waves in multiple physical dimensions such as phase, amplitude, and polarization by adjusting the microstructures [8,9,10,11]. The emergence of metasurface is providing new opportunities in many applications. Until now, metasurfaces have be broadly applied in the fields of wavefront shaping [12,13,14,15], holography [16,17,18], polarization modulation [19,20,21], optical communication [22,23,24], sensors [25,26,27], and so on. In general, metasurfaces can be divided into two main categories, metallic metasurfaces and dielectric metasurfaces, based on the primary class of the materials used [28,29]. The former exploits the surface plasmon oscillations; the latter relies on Mie resonance. Both kinds of metasurfaces have made considerable contributions to meta-optics, from spatial control to frequency mixing, in past decades. Compared to metallic metasurfaces, dielectric metasurfaces not only offer high transmission efficiency at visible and near-infrared frequency [30,31], but also have an easily tunable scattering owing to its dependence on geometry and high-input power threshold [32,33,34]. Moreover, the low costs of primary materials and compatibility with the state-of-art semiconductor process make dielectric metasurfaces a good choice for BS designs in photonic integration systems. Several metasurface-based BSs have already been presented in previous literature [35,36,37,38]. These BSs rely on the original polarization or frequency difference of the incident beam. Unfortunately, for applications utilizing polarized light status, there is still a lack of design with both polarization independence and adjustable power distribution. The BSs operating in arbitrary polarization with adjustable splitting ratio are in demand.

In this context, we present an all-dielectric metasurface as polarization-independent BS working at the near-infrared region. The gradient phase profiles encoded on metasurfaces are uniquely composed of silicon (Si) nano-rings on a fused quartz substrate. The simulations are based on Finite-Difference Time-Domain (FDTD). One period of the beam splitter consists of two Si nano-ring rows with opposite phase gradient of π/4. Different from the Pancharatnam–Berry phase nanoantennas working by rotating major axis and relying on polarization of incident beams, our design introduces field discontinuity along the interface. By spatially tailoring the inner and outer radiuses of the Si nano-rings, the phase gradient of the emergence beam can be flexibly controlled so that the wavefront phase changes regularly, forming the corresponding phase delay and resulting in the splitting of beams. The isotropy of nano-rings ensures the incident beams of any polarization will produce the same phase change. Furthermore, by changing the refractive index of the substrate or adding the Si film to the substrate, the splitting ratio of BS can be effectively regulated from 0.5:1 to 1:1. The power efficiency is over 92% and the total area of the device is only 33.6 μm × 33.6 μm, which can be easily integrated into optoelectronic systems and thus assists in the practical light path design for integrated optical communication, optical measurement, and so on.

## 2. Design Methodologies

**Figure 1:**
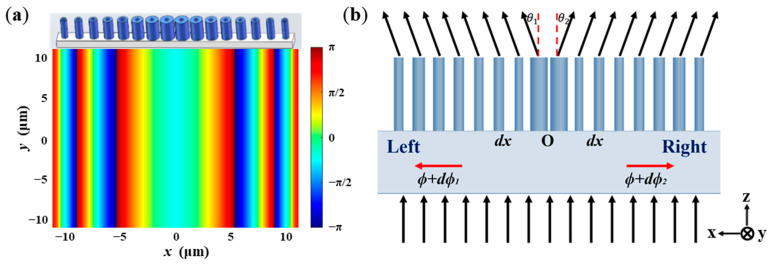
(**a**) The 3D structure and phase distribution of beam splitter design; (**b**) the schematic diagram of beam splitter orthograph.

The basic principle of designing metasurfaces is to introduce field discontinuity along the interface by spatially designing and arranging the geometry shape of the unit, which can control the wavefronts of the reflected or refracted beams. Figure 1 shows the schematic diagram of the all-dielectric metasurface-based BS. When the incident light field transmits through the Si nano-ring area, it will cause the effect of Fabry–Perot resonance, which will greatly change the effective length of the optical path and lead to different phase delays. The wavefront of the incident beam is divided into two parts from the center point O. According to the generalized Snell’s law, the emergence angles of $\theta_{1}$ and $\theta_{2}$ on the left and right beams can be expressed, respectively, by the following equations [39,40,41]:

(1) $$n_{t}\sin(\theta_{1})=n_{i}\sin(\theta_{i})-\frac{\lambda }{2\pi }\frac{d\phi_{1}}{dx},$$

(2) $$n_{t}\sin(\theta_{2})=n_{i}\sin(\theta_{i})+\frac{\lambda }{2\pi }\frac{d\phi_{2}}{dx},$$

where $\theta_{i}$ is the incident angle and $n_{i}$ and $n_{t}$ are the refractive index of the incident and refractive media respectively. $d\phi_{1}$ and $d\phi_{2}$ are the phase changes along the left and right sides, respectively. Suppose the phase gradient periods on the left and right side are $\Gamma_{1}$ and $\Gamma_{2}$. At the condition of normal incidence in air, the $\theta_{i}=0$ and $n_{i}=n_{t}$ Equations (1) and (2) can be written as:

(3) $$\sin(\theta_{1})=-\frac{\lambda }{\Gamma_{1}},$$

(4) $$\sin(\theta_{2})=\frac{\lambda }{\Gamma_{2}},$$

where Γ=p×l, p is lattice constant and l is the order of a phase gradient periods. From Equations (3) and (4), the emergence angles $\theta_{1}$ and $\theta_{2}$ are determined by the phase gradient periods $\Gamma_{1}$ and $\Gamma_{2}$ when the wavelength λ are fixed.

The splitting ratio of BS metasurface can be defined as:

(5) $$SR=\frac{T_{right}}{T_{left}},$$

Here, *T_left_* and *T_right_* represent the transmittance of the left and right sub-beams, respectively.

## 3. Simulation Results

### 3.1. The Transmittance and Phase Delay of the Structural Elements

**Figure 2:**
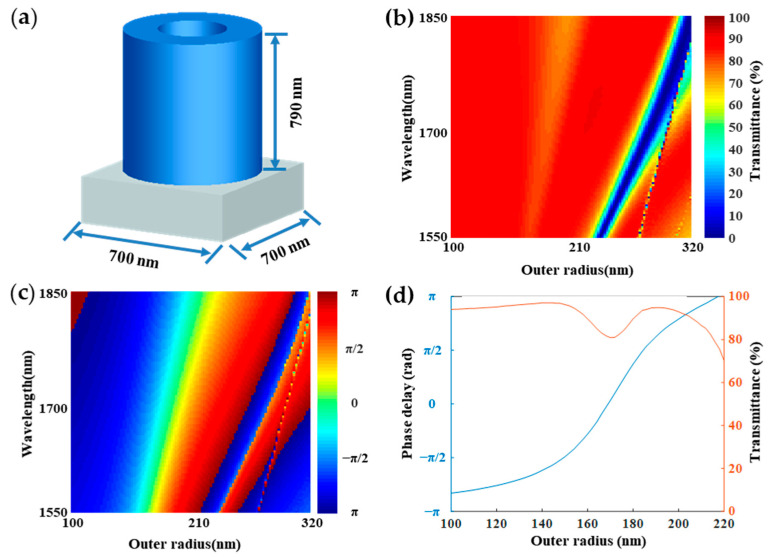
The properties of the beam splitter. (**a**) the element structure of the designed beam splitter based on a gradient metasurface; (**b**) the transmittance intensity of the nano-ring arrays in the infrared region; (**c**) the phase delay of the transmitted beam in the infrared region; (**d**) the resulting phase delay and transmittance of beam with different outer radius *R_out_* at the wavelength of 1550 nm.

Commercial software Lumerical FDTD Solution and Matlab were used for the metasurface simulation. Figure 2a shows the structure element of the BS. The Si nano-rings were arranged on a fused silica substrate, which works as building blocks with a lattice constant of *a* = 700 nm along the x and the y-axes. The height of the nano-ring was *h* = 790 nm. Here, we investigated the transmittance T and phase delay ϕ of the Si nano-rings with the wavelength from 1550 nm to 1850 nm. When the outer radius of nano-rings changed from 100 nm to 320 nm, and the inner radius was 30 nm, a plane wave propagated along the *z*-axis and polarized along the *x*-axis. The transmittance and phase delay of the Si nano-rings with different outer radius are shown in Figure 2b,c; it can be seen that the transmittance can be nearly regulated from 0 to 100% and the phase delay can be regulated from −π to π in the infrared region (from 1550 nm to 1850 nm), respectively. The phase delay and transmittance with various outer radius *R_out_* at the wavelength of 1550 nm are shown in Figure 2d. The phase delay ranging from 0 to −π is realized by adjusting the outer radius *R_out_* of the nano-rings from 100 nm to 218 nm with the transmittance over 80%. The result shows that as the outer radius increases, the phase delay increases. The silicon has a small loss in the infrared region, so the transmittance of the beam passing through the silica substrate is relatively high, and the transmittance is shown in Figure 2d [42,43].

**Figure 3:**
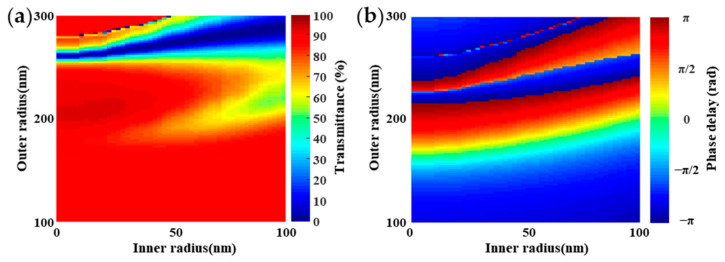
The transmittance T and the phase delay *ϕ* caused by the Si nano-rings with different combination of the outer and inner radiuses at the wavelength of 1550 nm. (**a**) The transmittance of the nano-ring arrays; (**b**) the phase delay of the nano-ring arrays.

**Figure 4:**
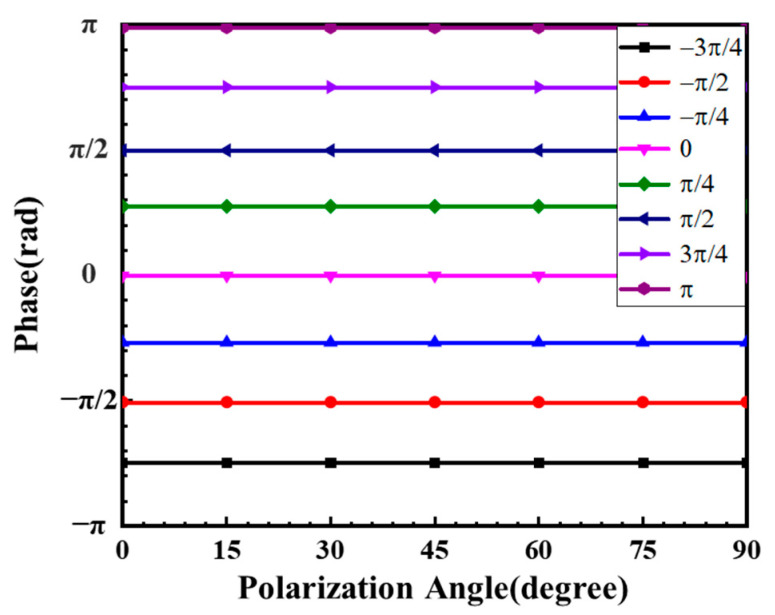
The verification of polarization independence for the nano-ring.

However, the small size difference among the outer radius *R_out_* will cause the instability and improve the difficulty of machining the device. To solve these problems, we changed both the inner radius and outer radius of nano-rings at the incident wavelength of 1550 nm. The transmittance T and the phase delay ϕ caused by the Si nano-rings are described in Figure 3. To achieve the desired phase delay and transmittance, the different combinations of the inner and outer radiuses have more selectivity, which is beneficial to improve the stability and reduce the machining difficulty. The transmittance and the phase delay with various inner and outer radiuses are shown in Table 1. It can be seen that the phase delay can be regulated from −π to π beginning with the point O with the transmittance over 92%. In order to demonstrate the polarization independence of the BS, the polarization direction of the incident beam was changed from 0 degrees (corresponding to horizontal polarization) to 90 degrees (corresponding to vertical polarization) in a step of 10 degrees. As shown in Figure 4, the nano-ring is almost insensitive to different linear polarization light. Due to the circular and elliptical polarization lights can be decomposed into two orthogonal linear polarization lights, the nano-ring is insensitive to polarization states. This is important for the sub-wavelength device, such as the BSs, which can identify the splitting ratio in different polarization direction.

### 3.2. The Construction of the Beam Splitter

**Figure 5:**
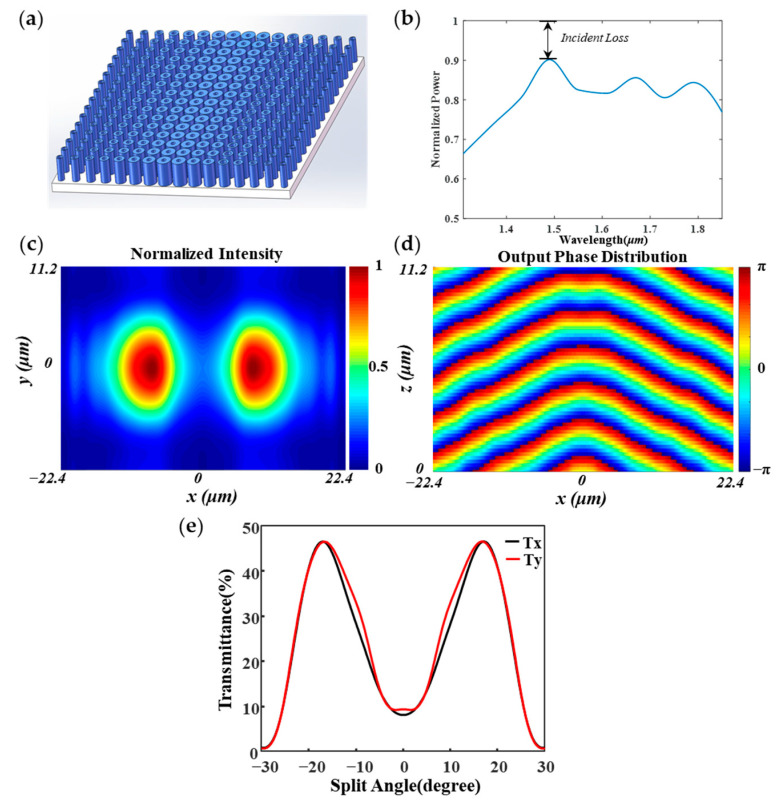
The properties of the nano-ring arrays. (**a**) 3D structure diagram. (**b**) Normalized power as function of wavelength from 1310 to 1850 nm. (**c**) The transmitted intensity distribution out of BS. (**d**) Simulated phase distribution propagate through the metasurface. (**e**) The transmittance curve of the BS.

According to our previous discussion, eight nano-rings with the phase gradient of π/4 are used to build the BS. One period structure of the BS is shown in Figure 5a. It is composed of two rows of Si-rings located on the left and right sides of the O point, and the phase delay gradually increases from left to right with phase gradient of π/4 on the left side and reverses on the right side. The element structure period of the BS is 5.6 μm in the *x*-axis and 0.7 μm in the *y*-axis. As shown in Figure 5b, the power efficiency of the metasurface array is above 0.5, from 1310 nm to 1850 nm wavelength. The full-width-half-maximum (FWHM) is around 500 nm, which shows that the beam splitter is suitable for the C-band and L-band range of the optics communication field. The incident loss (IL) has the lowest point at wavelength 1500 nm. The normalized intensity distribution of a plane wave propagating through the BS is shown in Figure 5c. Due to the existence of the phase gradient, the wavefront phase changes regularly, forming the corresponding phase delay, which leads to the splitting of the beam.

Furthermore, we used a Gaussian beam at the wavelength of 1550 nm to measure the splitting beam effect of the BS. The phase distributions of a plane wave propagating through the BS and transmittance curve are shown in Figure 5d,e, respectively. As shown in Figure 5c, the beam power is refracted to the left side and right side of the screen, and the presence of other diffraction orders with much smaller intensities compare to the main beam. As shown in Figure 5d, the black curve describes the typical result at the wavelength of 1550 nm under x polarization, and two refractive peaks appear at the emergence angles of 17.09° and −17.09°, which are close to theoretical predictions (16.07° and −16.07°) by Equations (3) and (4). The transmittances on the left and right side are 46.62% and 46.59%, which means that the splitting operation is almost realized. The splitting ratio is 1.00061 and the total transmission efficiency is 93.21%. To study the polarization independence of the BS, the Gaussian beam with y polarization was used as the incident beam. As shown in Figure 5d, the red curve shows two refractive peaks appearing at the emergence angles of 16.64° and −16.64°, which are also close to theoretical predictions. The transmittances on the left and right sides are 46.57% and 46.58%, the splitting ratio is 1.00035, and the total transmission efficiency is 93.15%. The result shows that the designed BS can be used to split beams with designated parameters, and the power efficiency is higher than 92%.

### 3.3. The Realization of Arbitrary Splitting Ratios

**Figure 6:**
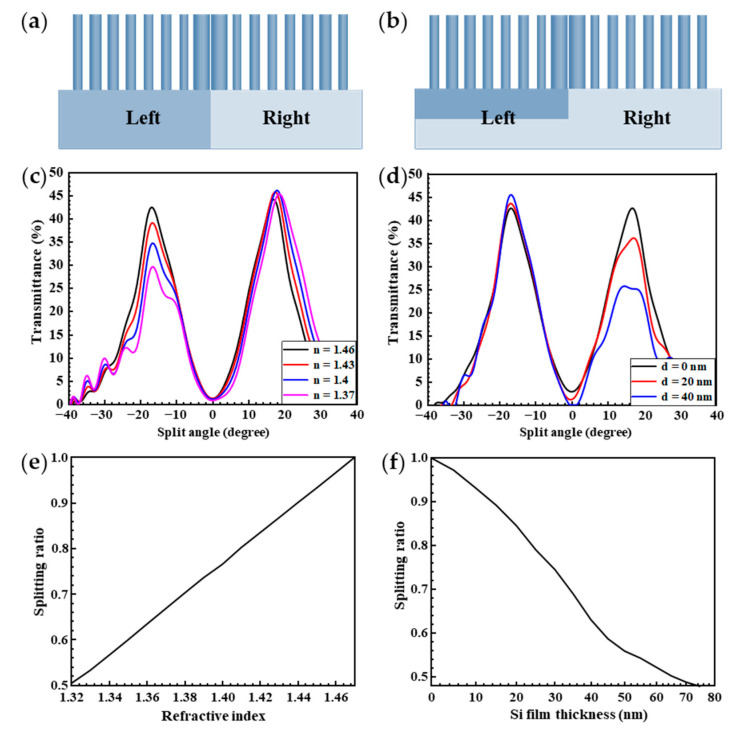
The realization for the arbitrary splitting ratio. (**a**) The structure diagram of the beam splitters, which the refractive index of the substrate on the left and right sides is different; (**c**,**e**) the corresponding transmittance and splitting ratio curve, respectively; (**b**) the structure diagram of the beam splitters with additional Si film in the left substrate; (**d**,**f**) the corresponding transmittance and splitting ratio curve, respectively.

The BS with arbitrary splitting ratios is crucial in the practical application of optics. By adjusting the refractive index of the left substrate or adding the Si film to the left substrate, we can easily control the splitting ratio of BSs. Figure 6a is the structure diagram of the BS, which the substrate refractive indexes on the left and right sides are different. The corresponding transmittance and splitting ratio curve are respectively depicted in Figure 6c,e. As shown in Figure 6c, the transmittance of left side gradually decreases while the other side remains. The corresponding splitting ratio is shown in Figure 6e; the splitting ratio increases from 0.5:1 to 1:1 with the increase of the refractive index of the substrate. This means that the splitting ratio can be adjusted by changing the refractive index of the substrate. Figure 6b is the other way that it can be used to adjust the splitting ratio by adding the Si film on the left substrate. Figure 6d,f are the corresponding transmittance and splitting ratio curve, respectively. As shown in Figure 6d, the transmittance of the right side gradually decreases while the other is retained. This means that the splitting ratio can be adjusted by adding the Si film with different thickness to the left substrate. For the corresponding splitting ratios, as shown in Figure 6f, the splitting ratio decreases from 1:1 to 0.45:1 with the increase of Si film thickness on the left substrate. This means that the splitting ratio can be adjusted by adjusting the refractive index of the substrate or adding the Si film with a different thickness to the substrate; this is because changing the refractive index or adding the Si film to the substrate does not affect the phase gradient but can affect the initial phase of the incident beams. In sum, we understand that the splitting ratio can be adjusted from nearly 0.5:1 to 1:1 by changing the refractive index of the substrate or adding the Si film with different thickness.

**Figure 7:**
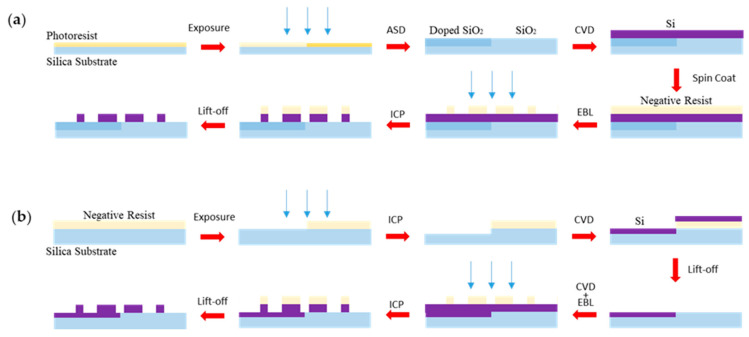
The assumed fabrication process of arbitrary splitting ratios beam splitter. (**a**) Changing refractive index of the substrate. (**b**) Adding the Si film to the substrate.

In our tentative idea, the change of the metasurface splitting ratio is based on the multiple exposure and area-selective deposition (ASD) fabrication technique. The assumed fabrication process of the refractive index change in Figure 6a is shown in Figure 7a. The silica substrate is fist spin coated with photoresist. The patterns are transferred from photomasks to photoresists after the exposure and development steps, which create two regions with different properties. The ASD technique refers to any chemical or physical process that controllably forms a desired material layer on a desired “growth” region of an exposed surface without forming a layer on other adjacent “non-growth” areas of different compositions or surface terminations [44,45]. The growth and non-growth regions can be differentiated by material composition, surface termination, lattice structure, or physical topography. In the process of ASD, the doped SiO_2_ with designed refractive index are deposited in the left region while the regular SiO_2_ are in the right side. Then, a Si film grows on the top of the whole region by chemical vapor deposition (CVD). After spin coating a layer of negative resist on the sample, electron-beam lithography (EBL) directly draws the ring patterns with nanoscale featured sizes and is followed by induced coupled plasma (ICP) silicon etching. Finally, when the remaining resist is cleared by the lift-off process, the designed beam splitter metasurface with different refractive index is completed. Similarly, the assumed fabrication process of the Si film change in Figure 6b are shown in Figure 7b. After double exposure, etching, depositing, and lift-off process, the silica substrate can obtain certain Si film in the specified region.

## 4. Discussion

The wavefront phase of the emergence beam can be adjusted to form a phase gradient to split beams. We have demonstrated that the wavefront phase of any polarized light can be formed by the nano-ring arrays with designed phase gradient. The splitting ratio of the BS can be adjusted by changing the properties of the substrate. However, there are still some limitations, such as the splitting ratio and that the emergence angle of the BS is not tunable in working condition and their adjustable range is narrow. Thus, we need to improve existing designs and explore new types of materials to overcome these limitations. For example, two-dimensional materials are able to dynamically adjust refractive index by external applied fields. The metasurface-based BSs will be expected to work better with other silicon-based devices on a unified platform. They are promising for integrated optics applications such as free space optical communication, optical measurement, biological photonics, etc.

## 5. Conclusions

In this paper, we proposed and designed an all-dielectric metasurface-based and polarization-independent BS in the near-infrared region, which has advantages of small size, higher power efficiency, and a regulated splitting ratio. The wavefront phase of the emergence beam can be adjusted to form a phase gradient to split beams. The proposed beam splitter exhibits the desirable abilities of separating an incident light of any polarization toward two pre-designed directions. The splitting ratio of the BSs in any desired proportion can be realized by adjusting the refractive index of the substrate or adding the Si film; this is because changing the refractive index or adding the Si film will not affect the phase gradient but affect the initial phase of incident beams. In the result, its splitting ratio can be regulated from 0.5:1 to 1:1 and the power efficiency reaches over 92% with total area of 33.6 μm × 33.6 μm. Compared to conventional bulky optical components, this metasurface-based beam splitter can be assembled into the photonic integrated optical systems of applications.

## Figures and Tables

**Table 1:** The transmittance and phase delay with the various inner and outer radiuses.

***R_out_* (nm)**	216	144	150	160	168	172	180	196	214
***R_in_* (nm)**	22	94	48	26	28	12	12	28	8
**Phase**	−π	−3π/4	−π/2	−π/4	0	π/4	π/2	3π/4	π
***T* (%)**	99.88	96.83	96.69	96.112	93.56	93.20	92.82	96.35	99.48

## Data Availability

Data is contained within the article.
